# Supplementary material for: High-precision targeting workflow for volume electron microscopy
Source: J Cell Biol. 2021 Jun 23;220(9):e202104069. doi: 10.1083/jcb.202104069 (PMC8225610; doi:10.1083/jcb.202104069)
Supplement: Table S1 — summarizes the FS protocol (Leica AFS2 with FSP unit). [file JCB_202104069_TableS1.docx]

**Table S1. Freeze substitution protocol (Leica AFS2 with FSP unit)**

| **Step #** | **Tstart** | **Tend** | **Slope** | **Time** | **Solution** | **Operation** | **Agitation** | **UV** |
| --- | --- | --- | --- | --- | --- | --- | --- | --- |
| **1** | -90°C | -90°C | 0°C/h | 72h | FS cocktail | stay |  |  |
| **2** | -90°C | -45°C | 3°C/h | 15h | FS cocktail | stay |  |  |
| **3** | -45°C | -45°C | 0°C/h | 5h | FS cocktail | stay |  |  |
| **4** | -45°C | -45°C | 0°C/h | 10min | acetone | exchange/fill |  |  |
| **5** | -45°C | -45°C | 0°C/h | 10min | acetone | exchange/fill |  |  |
| **6** | -45°C | -45°C | 0°C/h | 10min | acetone | exchange/fill |  |  |
| **7** | -45°C | -45°C | 0°C/h | 6h | Lowicryl 10% | mix | on |  |
| **8** | -45°C | -45°C | 0°C/h | 6h | Lowicryl 25% | mix | on |  |
| **9** | -45°C | -35°C | 1.67°C/h | 6h | Lowicryl 50% | mix | on |  |
| **10** | -35°C | -25°C | 1.67°C/h | 6h | Lowicryl 75% | mix | on |  |
| **11** | -25°C | -25°C | 0°C/h | 10h | Lowicryl 100% | exchange/fill |  |  |
| **12** | -25°C | -25°C | 0°C/h | 10h | Lowicryl 100% | exchange/fill |  |  |
| **13** | -25°C | -25°C | 0°C/h | 10h | Lowicryl 100% | exchange/fill |  |  |
| **14** | -25°C | -25°C | 0°C/h | 48h | Lowicryl 100% | stay |  | on |
| **15** | -25°C | +20°C | 5°C/h | 9h | Lowicryl 100% | stay |  | on |
